# The Metalloproteinase ADAM28 Promotes Metabolic Dysfunction in Mice

**DOI:** 10.3390/ijms18040884

**Published:** 2017-04-21

**Authors:** Lakshini Herat, Caroline Rudnicka, Yasunori Okada, Satsuki Mochizuki, Markus Schlaich, Vance Matthews

**Affiliations:** 1 Dobney Hypertension Centre, School of Medicine and Pharmacology, University of Western Australia, Crawley WA 6009, Australia; lakshini.weerasekera@uwa.edu.au (L.H.); markus.schlaich@uwa.edu.au (M.S.); 2 Research Centre, Royal Perth Hospital, Perth WA 6000, Australia; caroline.rudnicka@uwa.edu.au; 3 Department of Pathophysiology for Locomotive and Neoplastic Diseases, Juntendo University Graduate School of Medicine, Tokyo 113-8421, Japan; ya-okada@juntendo.ac.jp; 4 Department of Surgery, National Defense Medical College, Saitama 359-8513, Japan; s-mochi@ndmc.ac.jp; 5 Department of Cardiology and Department of Nephrology, Royal Perth Hospital, Perth WA 6000, Australia

**Keywords:** ADAM28, type 2 diabetes (T2D), obesity, metabolic syndrome, metalloproteinases

## Abstract

Obesity and diabetes are major causes of morbidity and mortality globally. The current study builds upon our previous association studies highlighting that A Disintegrin And Metalloproteinase 28 (ADAM28) appears to be implicated in the pathogenesis of obesity and type 2 diabetes in humans. Our novel study characterised the expression of ADAM28 in mice with the metabolic syndrome and used molecular inhibition approaches to investigate the functional role of ADAM28 in the pathogenesis of high fat diet-induced obesity. We identified that ADAM28 mRNA and protein expression was markedly increased in the livers of mice with the metabolic syndrome. In addition, noradrenaline, the major neurotransmitter of the sympathetic nervous system, results in elevated *Adam28* mRNA expression in human monocytes. Downregulation of ADAM28 with siRNA technology resulted in a lack of weight gain, promotion of insulin sensitivity/glucose tolerance and decreased liver tumour necrosis factor-α (TNF-α) levels in our diet-induced obesity mouse model as well as reduced blood urea nitrogen, alkaline phosphatase and aspartate aminotransferase. In addition, we show that ADAM28 knock-out mice also displayed reduced body weight, elevated high density lipoprotein cholesterol levels, and reductions in blood urea nitrogen, alkaline phosphatase, and aspartate aminotransferase. The results of this study provide important insights into the pathogenic role of the metalloproteinase ADAM28 in the metabolic syndrome and suggests that downregulation of ADAM28 may be a potential therapeutic strategy in the metabolic syndrome.

## 1. Introduction

Obesity is one of the most prevalent metabolic diseases globally and is an established risk factor for type 2 diabetes (T2D). Excess weight correlates directly with glucose intolerance and insulin resistance, which may ultimately lead to the development of T2D [1]. Recent studies have identified associations between obesity and T2D involving pro-inflammatory cytokines (tumour necrosis factor-α (TNF-α) and interleukin-6), insulin resistance, deranged fatty acid metabolism, and cellular processes such as mitochondrial dysfunction and endoplasmic reticulum stress [2]. Risk factors for obesity and fatty liver disease may include consumption of diets high in fat and fructose [3,4,5] and low physical activity [6].

A Disintegrin And Metalloproteinases, or ADAMs, are a group of transmembrane and secreted proteins which play an important role in regulating cell phenotype via their effects on cell adhesion, migration, proteolysis, and signalling [7]. These proteins have a major impact on the pathogenesis of numerous diseases. The metalloproteinase ADAM28, also known as lymphocyte metalloprotease MDC-L, was first identified on lymphoid cells and has two isoforms: (i) a membrane-type form (ADAM28m) and (ii) a secreted soluble form (ADAM28s). A number of early studies suggested that ADAM28 may be important in inflammation and metabolism [8,9]. One known substrate for ADAM28 is the pro-inflammatory cytokine TNF-α. Interestingly, a number of synthetic peptides containing the authentic TNF-α shedding site were shown to be cleaved by ADAM28 [8]. Our recent studies have confirmed that human ADAM28 is a novel sheddase of human TNF-α [9] and reinforced the notion that ADAM28 is a novel sheddase of one of the major pro-inflammatory cytokines involved in the pathogenesis of the metabolic syndrome.

In our current study, we aimed to translate our previous findings [9] into an in vivo animal model to assess the effects of limiting ADAM28 activity on parameters of the metabolic syndrome. In this study, we establish that ADAM28 is pathogenic in the metabolic syndrome and provide evidence that metalloproteinase inhibition is a potential therapeutic target for anti-obesity agents.

## 2. Results

**Figure 1:**
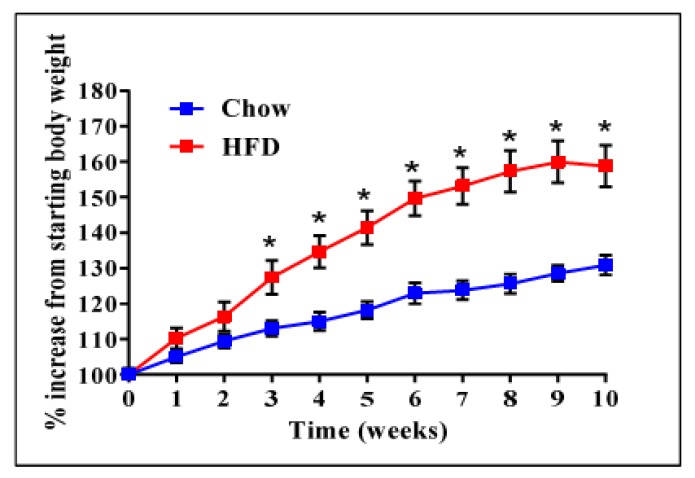
High fat diet (HFD) induced obesity in mice before small interfering RNA (siRNA) administration. *n* = 9 mice/group, * *p* < 0.05.

We have previously reported that high expression of ADAM28 mRNA in peripheral blood mononuclear cells from the San Antonio Family Heart Study (SAFHS) cohort (*n* = 1240) correlated strongly with parameters of the metabolic syndrome [9]. To further our previously published findings, we conducted ADAM28 expression and functional studies in our murine high fat diet-induced obesity model. Mice were weighed weekly to confirm obesity (Figure 1). High fat diet fed mice were glucose intolerant and insulin resistant compared to their chow fed counterparts, as previously reported [10]. In addition, livers of high fat diet fed mice were markedly steatotic with inflammatory cell infiltration, as demonstrated in our previous study [10].

### 2.1. ADAM28 mRNA Expression Is Significantly Elevated in the Liver of High Fat Diet Fed Mice

**Figure 2:**
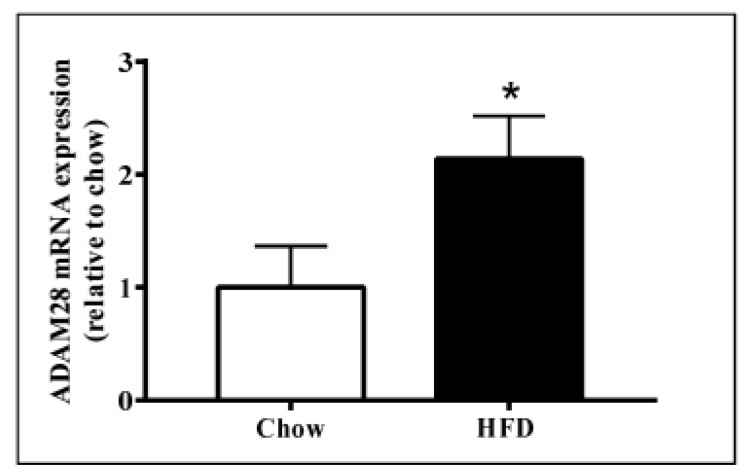
Increased A Disintegrin And Metalloproteinase 28 (ADAM28) mRNA expression in livers of mice fed a high fat diet (HFD). Expression of ADAM28 mRNA in livers from mice fed either a normal chow or high fat diet. * *p* < 0.05; *n* = 12–14.

**Figure 3:**
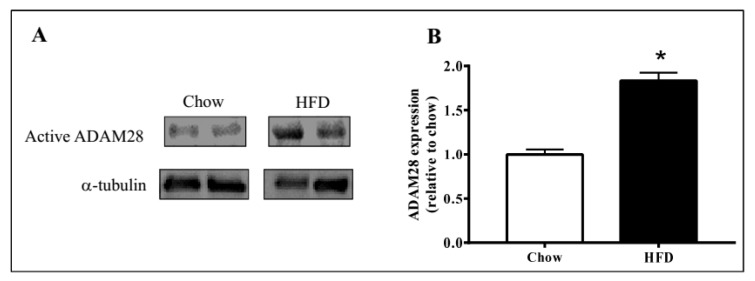
Elevated ADAM28 expression levels in high fat fed mice. Active ADAM28 (42 kDa) protein levels were measured by immunoblotting (**A**) and quantified based on densitometry (**B**) in livers of mice on a high fat diet for 12 weeks * *p* < 0.05; *n* = 3–4 mice/group.

ADAM28 mRNA and protein expression in the liver was studied, as the liver is an organ well known for lipid and glucose metabolism [11]. We demonstrated that ADAM28 mRNA levels are increased in the livers of mice fed a high fat diet for 12 weeks (Figure 2). In addition, active ADAM28 (42 kDa) protein levels were also increased in the livers of mice fed a high fat diet, as evidenced by western blotting (Figure 3).

### 2.2. ADAM28 Expression Is Elevated in Human Monocytes Treated with Noradrenaline (NA)

**Figure 4:**
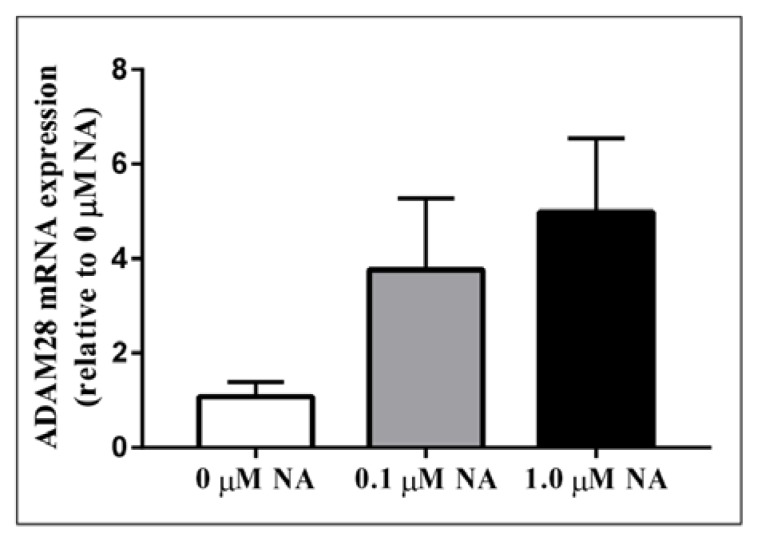
Noradrenaline treatment of human THP-1 monocytes promotes elevated ADAM28 mRNA expression. Cells were treated for 48 h.

Activation of the sympathetic nervous system (SNS) is a cardinal feature of obesity, metabolic syndrome, and type 2 diabetes (T2DM) and is associated with disease progression [12]. In order to test our hypothesis that sympathetic nervous system activation may result in elevated ADAM28 expression, we treated human THP-1 monocytes with noradrenaline (NA), the main neurotransmitter of the SNS. Excitingly, we have now shown for the first time that NA treatment may result in elevated ADAM28 expression in a dose-dependent manner (Figure 4). The difference between 0 and 1.0 µM NA treatment groups was close to reaching significance (*p* = 0.0698).

### 2.3. In Vivo Knock-Down of ADAM28 Ameliorated Parameters of the Metabolic Syndrome

**Figure 5:**
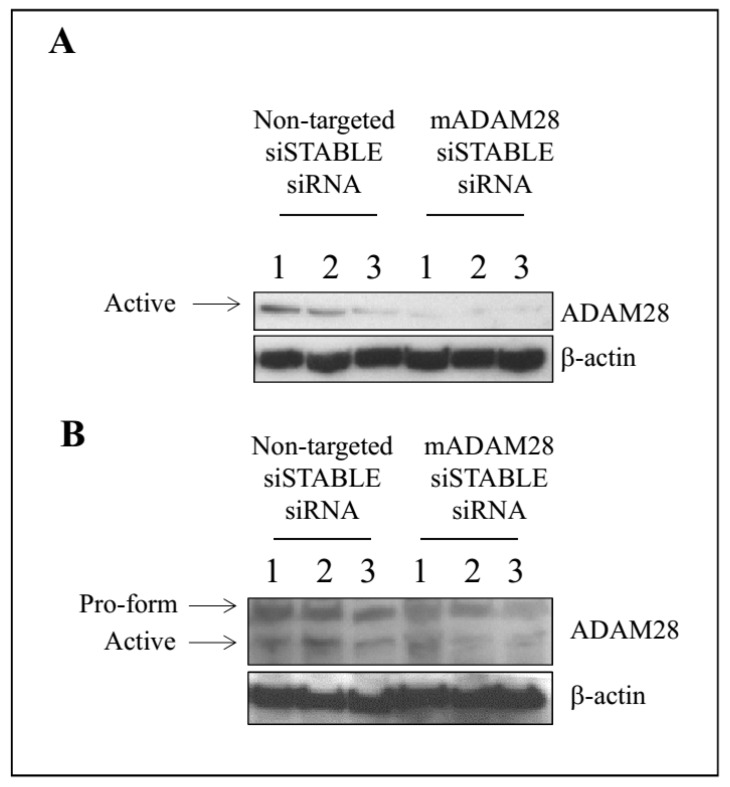
In vivo knock-down of ADAM28 in liver (**A**) and white adipose tissue (**B**) of high fat diet fed mice. The siRNA was administered for the final 2 weeks of the diet (*n* = 3 mice/group).

A vast array of studies have highlighted the ability of siRNA therapy to improve numerous disease states [13,14,15,16]. We have previously shown our capacity to successfully knock-down ADAM protein expression utilising siRNA technology [17]. Our next aim was therefore to utilise siRNA targeting mouse ADAM28 to reduce ADAM28 expression in vivo in our high fat diet-induced obesity mouse model. Our results demonstrate successful reduction of ADAM28 protein expression in the liver of mice treated with siRNA targeting mouse ADAM28 (mADAM28 siSTABLE siRNA) (Figure 5A). Interestingly, only the active form of ADAM28 was detected in the liver, suggesting all pools of ADAM28 are activated in the liver. The pro-form and active form of ADAM28 are observed in gonadal white adipose tissue (Figure 5B). Infiltrating inflammatory cells may be contributing to this expression in gonadal white adipose tissue. ADAM28 protein levels were mildly decreased in white adipose tissue of mice treated with ADAM28 siRNA.

**Figure 6:**
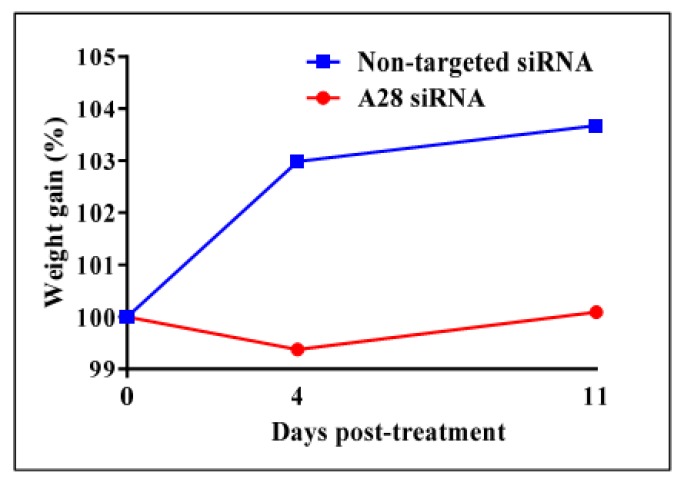
In vivo knock-down of mouse ADAM28 reduces high fat diet-induced mean weight gain. (*n* = 3 mice/group).

**Figure 7:**
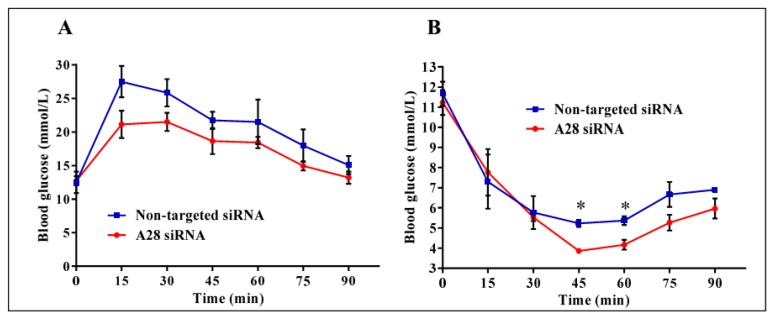
Metabolic testing in mice treated with ADAM28 siRNA. In vivo knock-down of ADAM28 reduces high fat diet-induced glucose intolerance (**A**), as demonstrated with glucose tolerance testing, and insulin resistance (**B**), as demonstrated with insulin tolerance testing in mice. * *p* < 0.05; (*n* = 3 mice/group).

**Figure 8:**
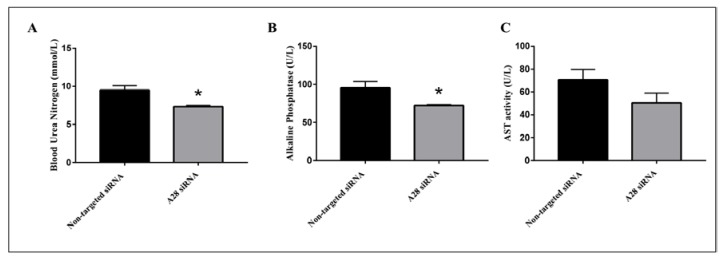
Metabolic benefits in mice treated with ADAM28 siRNA. Blood urea nitrogen (**A**); Alkaline Phosphatase (**B**); and aspartate aminotransferase (AST) activity (**C**). * *p* < 0.05; *n* = 3 mice/group.

**Figure 9:**
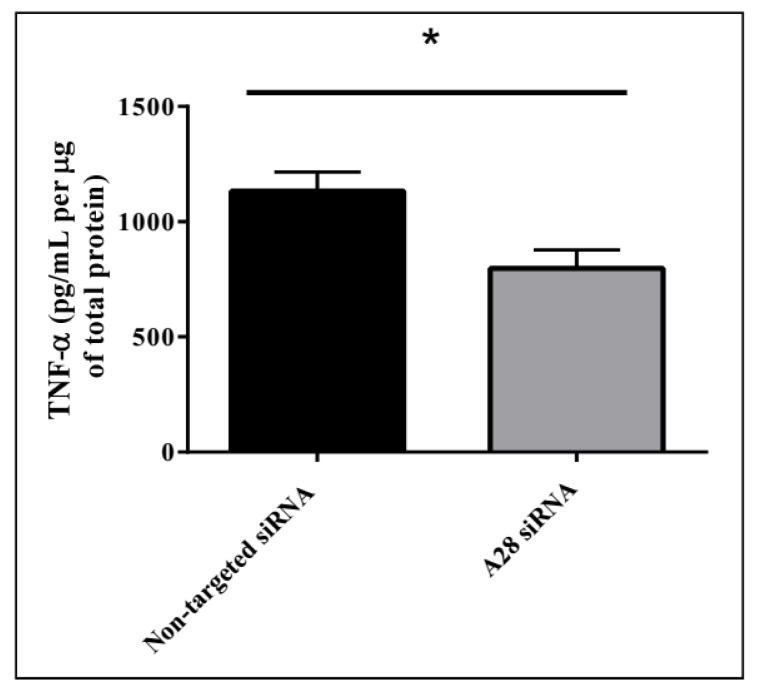
Decreasing ADAM28 expression reduces liver TNF-α levels in high fat diet (HFD) fed mice. Mean + SEM; * *p* < 0.05; *n* = 3 mice/group.

Mice treated with mADAM28 siSTABLE siRNA also showed improvements in several parameters of the metabolic syndrome including the failure to exhibit further increases in high fat diet-induced weight gain compared to mice treated with control siRNA (non-targeted siSTABLE siRNA) (Figure 6). In addition, mADAM28 siSTABLE siRNA treated mice displayed increased glucose tolerance (Figure 7A) and insulin sensitivity (Figure 7B) compared to mice treated with control siRNA. In addition, blood urea, which is indicative of kidney function was reduced in the serum of ADAM28 siRNA treated mice (Figure 8A), suggesting that kidney function may be better preserved in ADAM28 siRNA treated mice. Liver enzymes, which are indicative of liver injury, such as alkaline phosphatase (Figure 8B) and aspartate aminotransferase (AST) (Figure 8C) were markedly reduced in the serum of ADAM28 siRNA treated mice. As ADAM28 is a sheddase of TNF-α protein, we measured the TNF-α protein levels by ELISA in the livers of mice treated with either non-targeted siRNA or ADAM28 siRNA. We found that reducing ADAM28 activity resulted in reduced TNF-α protein levels in the liver (Figure 9). The TNF-α protein may be cleaved or cytoplasmic derived TNF-α.

### 2.4. Metabolic Benefits in ADAM28 Knock-Out (KO) Mice

**Figure 10:**
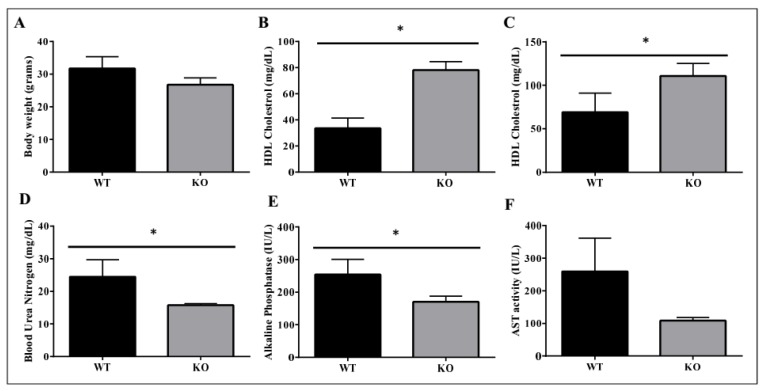
Metabolic benefits in female ADAM28 knock-out (KO) mice. Body weight at 10 months of age (**A**); High-density lipoprotein (HDL) cholesterol at 49 days of age (**B**); HDL cholesterol at 6 months of age (**C**); Blood urea nitrogen at 49 days of age (**D**); Alkaline Phosphatase at 49 days of age (**E**) and AST activity at 49 days of age (**F**). * *p* < 0.05; *n* = 3–4 mice/group.

We used ADAM28 knock-out (KO) mice to determine if the absence of ADAM28 promoted metabolic benefits. In particular, ADAM28 KO mice are viable as adults with a normal life span. Excitingly, in agreement with our hypothesis, ADAM28 KO mice on a normal chow diet possess a reduced body mass at 10 months of age (Figure 10A; *p* = 0.05335). Serum high-density lipoprotein (HDL) cholesterol levels were significantly elevated in ADAM28 KO mice at 49 days (Figure 10B) and 6 months of age (Figure 10C). Blood urea, which is indicative of kidney function, was reduced in the serum of ADAM28 KO mice (Figure 10D), suggesting that kidney function may be better preserved in ADAM28 KO mice. Liver enzymes, which are indicative of liver injury, such as alkaline phosphatase (Figure 10E) and aspartate aminotransferase (AST) (Figure 10F), were markedly reduced in the serum of ADAM28 KO mice at 49 days of age.

## 3. Discussion

For the first time, we have examined the role of ADAM28 in the metabolic syndrome in an in vivo mouse model. We found ADAM28 mRNA and protein levels to be higher in steatotic livers of obese mice. In addition, noradrenaline, the major neurotransmitter of the sympathetic nervous system, results in elevated *Adam28* mRNA expression in human monocytes. Using siRNA technology, we also demonstrated that downregulation of ADAM28 resulted in a lack of weight gain, promotion of insulin sensitivity/glucose tolerance and decreased liver TNF-α levels in our diet-induced obesity mouse model as well as improved kidney function, and reduced liver injury . Our study also highlighted the metabolic benefits in ADAM28 knock-out mice. 

An increasing number of studies suggest that ADAM28 plays a crucial role in the pathogenesis of several diseases [18,19,20,21,22,23,24,25], particularly in cancers such as breast cancer [19], prostate cancer [21], B-cell acute lymphoblastic leukaemia [25], chronic lymphatic leukaemia [23], head and neck squamous cell carcinoma [24] and other conditions such as lethal acute respiratory infections [18]. However, the role of ADAM28 in colorectal cancer remains controversial [20,22]. Here, we provide evidence to show in our current functional study that ADAM28 appears to also play a pathogenic role in the metabolic syndrome.

It is known that ADAM28 is expressed in immune cells in mice and humans, primarily in the B-lymphocyte lineage [26,27]. It would be interesting to conduct future bone marrow transplantation studies using ADAM28 KO bone marrow to ascertain the role that ADAM28 expression in cells of the hematopoietic lineage has in the metabolic syndrome and the aforementioned diseases.

Indeed, there is increasing evidence that obesity and T2D are associated with a chronic inflammatory state and the important role of metalloproteinases in this inflammatory paradigm is being increasingly recognized [9,28,29]. We have previously documented several mechanisms by which the metalloproteinase and disintegrin domains of ADAM28 may promote inflammation and ultimately metabolic dysfunction [9]. Our group has highlighted that major substrates of the metalloproteinase domain of ADAM28 include IGFBP-3 and TNF-α which may confer adipogenesis and inflammation, respectively [9,30]. Hence, based on our current study, it is plausible that therapeutic ADAM28 inhibition may reduce adipogenesis and inflammation due to diminished IGFBP-3 cleavage and TNF-α shedding. We did indeed demonstrate that in our in vivo studies in HFD-fed mice that silencing ADAM28 expression resulted in a marked reduction in TNF-α protein in the liver. This TNF-α protein may be cleaved or cytoplasmic derived.

Our previous work [9] and that from other groups [20] have reported that ADAM28 is elevated in overweight and obese humans and correlates with several parameters of the metabolic syndrome. The results in our present in vivo mouse study indicated that siRNA mediated downregulation of ADAM28 promoted decreased high fat diet-induced weight gain, increased glucose tolerance/insulin sensitivity, decreased liver TNF-α levels, improved kidney function, and reduced liver injury. We also illustrate in ADAM28 knock-out mice that body weight is decreased, levels of protective high density lipoprotein cholesterol are significantly elevated, whilst kidney function is better preserved and liver injury is reduced. Therefore, our current data further supports ADAM28’s pathogenic role in the metabolic syndrome. Future studies should address the effect of ADAM28 expression on leptin levels, as leptin plays numerous beneficial metabolic roles such as appetite control [31,32].

There are currently numerous physical, clinical, and therapeutic strategies to manage obesity and obesity related disorders such as type 2 diabetes. These strategies include physical activity [6], bariatric surgery [33,34], metformin therapy [35], consumption of a Mediterranean diet [36,37], natural products [38] and herbal medications [39].

Reducing ADAM28 levels with siRNA technology resulted in no gains in body weight, increased glucose tolerance/insulin sensitivity, decreased liver TNF-α levels, improved kidney function, and reduced liver injury. The siRNA approach would reduce all domains of the ADAM28 protein, including the disintegrin domain. The disintegrin domain may be critically involved in the pathogenic role of ADAM28. We have previously discussed how binding of the disintegrin domain of ADAM28 to integrin α4β1 and/or P-selectin glycoprotein ligand-1 on leukocytes may promote inflammation [9].

Our novel findings show that increased ADAM28 mRNA and protein expression in high fat diet-induced obesity is associated with promoting features of the metabolic syndrome in mice. Additionally, ablation of ADAM28 in mice on normal chow confers metabolic benefits. These results provide evidence that downregulation of ADAM28 could be a potential therapeutic target for anti-obesity agents.

## 4. Materials and Methods

### 4.1. Cell Culture Experiments

THP-1 human monocyte cells (<6 passages) were purchased from the American Type Culture Collection (Manassas, VA, USA). Cells were cultured at 37 °C, 5% CO_2_ in a humidified chamber. THP-1 cells were cultured in Dulbecco’s Modified Eagle Medium (DMEM) [high glucose; Gibco, Gaithersburg, MD, USA] + 5% foetal calf serum (FCS) and 1% penicillin/streptomycin. For noradrenaline treatments, cells were washed and changed to starvation media (High glucose DMEM + 1.0% FCS). Cells were treated with 0, 0.1, or 1.0 μM noradrenaline (Sigma, St. Louis, MO, USA) for 48 h.

### 4.2. Animals

All experimental and animal handing activities were performed in accordance with the guidelines of the Institutional Animal Care and Use Committee of the Royal Perth Hospital, Western Australia. Animal ethics approval (#R522/13–16) for our experiments was received from the Royal Perth Hospital Animal Ethics Committee. Eight-week-old male specific pathogen free C57BL6/J mice were obtained from the Animal Resources Centre (ARC, Perth, WA, Australia). Mice were acclimatized for 7 days, housed under a 12-h light/dark cycle, and given a standard diet with free access to food and water.

In our first experiments, mice were administered a normal chow diet (14.3 MJ/kg, 76% of energy from carbohydrate, 5% from fat, 19% from protein; Specialty Feeds, Glen Forrest, WA, Australia) or high fat diet, HFD (19 MJ/kg, 35% of energy from carbohydrate, 42% from fat, 23% from protein; Speciality Feeds, Glen Forrest, WA, Australia) for 12 weeks, and body weights were recorded weekly. At the end of the experiment, mice were sacrificed and livers were collected for paraffin embedding and snap frozen in liquid nitrogen for mRNA studies.

### 4.3. ADAM28 siSTABLE siRNA Treatment 

Eight-week-old male specific pathogen free C57BL6/J mice were placed on different diet/antibody treatment regiments: (1) Standard chow: administered non-targeted siSTABLE siRNA (*n* = 3); (2) Standard chow: administered siSTABLE mouse ADAM28 siRNA (*n* = 3); (3) High fat diet: administered non-targeted siSTABLE siRNA (*n* = 3); and (4) High fat diet: administered siSTABLE mouse ADAM28 siRNA (*n* = 3). The siRNA administration occurred at the end of week ten of the dietary regiment, as ADAM28 mRNA is increased at this time-point in tissues such as the liver. Mice received siRNA injections every five days via the tail vein for the final two weeks of feeding. For each time-point, 20 µg of siSTABLE siRNA (Dharmacon, Lafayette, CO, USA) was mixed with 200 µL DOTAP liposomal transfection reagent (Roche, Indianapolis, IN, USA). Body weight measurements were collected weekly. Glucose tolerance tests were performed at the start of week 12 and insulin tolerance tests were performed at the end of week 12, as indicated previously [10]. Liver and adipose tissue were collected and snap frozen. Blood urea nitrogen, alkaline phosphatase, and aspartate aminotransferase were measured in serum by PathWest LMWA (Murdoch, WA, Australia).

### 4.4. Western Blotting

Liver and gonadal white adipose tissue were homogenised in cytosolic extraction buffer containing phosphatase and protease inhibitors. Protein levels were determined using a Bradford protein assay. Cell lysates were cleared and protein concentration calculated using protein assay solution (Bio-Rad, Hercules, CA, USA). Protein lysates were solubilized in Laemmli sample buffer and boiled for 10 min, resolved by SDS–polyacrylamide gel electrophoresis on 10% polyacrylamide gels, transferred by semi-dry transfer to polyvinylidene difluoride membrane, and then blocked with 5% milk powder. Membranes were then incubated overnight at 4 °C in anti-mouse ADAM28 monoclonal antibody (sc-514228 [H4], Santa Cruz Biotechnology, Paso Robles, CA, USA); α-tubulin (Santa Cruz Biotechnology; sc-5546) or β-actin (Abcam, Cambridge, UK; ab6276) using recommended dilutions. Membranes were washed three times in washing buffer and incubated for 60 min at room temperature with either anti-rabbit or anti-mouse horse-radish peroxidase (HRP; GE, Issaquah, WA, USA), respectively. Membranes were then washed and briefly incubated in Amersham ECL Prime Western Blotting Detection Reagent (GE, Issaquah, WA, USA). The protein bands were detected using the Alpha Innotech MultiImage II Fluor Chem FC2 (Miami, FL, USA).

### 4.5. Adam28 mRNA Expression Studies in Human THP-1 Monocytes and Livers of Mice Fed Normal Chow or High Fat Diet (HFD)

RNA from human THP-1 monocytes and livers of mice (fed normal chow or HFD) was extracted using Trizol reagent (Invitrogen, Carlsbad, CA, USA) and cDNA synthesis was performed using the High Capacity RNA-to-cDNA kit (Thermofisher Scientific, Waltham, MA, USA). Real-time PCR to determine the mRNA abundance of human or mouse *Adam28* and *18S rRNA* (house-keeper gene) was performed using a Rotor-gene real-time PCR machine (Qiagen, Germantown, MD, USA) using pre-developed TaqMan probe and primer sets for human *Adam28* (Hs00248020_m1), human *18S* (Hs03928990_g1), mouse *Adam28* (Mm00456637_m1), and eukaryotic *18S rRNA* (4310893E) (Thermofisher Scientific, Waltham, MA, USA). Quantitation was conducted as previously described [40].

### 4.6. ADAM28 KO Mice

An Academic Delta One licence agreement was obtained from Deltagen (San Mateo, CA, USA) to access phenotypic data for female ADAM28 knock-out (KO) mouse fed a normal chow diet (t137). Permission to publish the data was obtained from Robert Driscoll.

### 4.7. TNF-α ELISA on Murine Liver Protein

Liver tissue was homogenised in cytosolic extraction buffer containing phosphatase and protease inhibitors. Protein levels were determined using a Bradford protein assay and TNF-α was measured in lysates using a mouse TNF-α ELISA (ELISAkit.com, Caribbean Park, Scoresby, VIC, Australia).

### 4.8. Statistics

All in vitro and in vivo results are expressed as the mean + and/or − standard error of the mean (SEM). Data were analysed for differences by Students *t*-test for unpaired samples where appropriate. Data was considered to be statistically significant when *p* < 0.05. T values were also calculated to further verify significance (Table S1).

## Supplementary Materials

Supplementary materials can be found at http://www.mdpi.com/1422-0067/18/4/884/s1.

## Abbreviations

ADAM28	A Disintegrin And Metalloproteinase 28
AST	Aspartate aminotransferase
DMEM	Dulbecco’s Modified Eagle Medium
FCS	Foetal Calf Serum
HDL	High Density Lipoprotein
HFD	High fat diet
KO	Knock-out
mRNA	Messenger RNA
NA	Noradrenaline
SAFHS	San Antonio Family Heart Study
SEM	Standard error of the mean
siRNA	Small interfering RNA
SNS	Sympathetic nervous system
T2D	Type 2 Diabetes
TNF-α	tumour necrosis factor-α
