# Supplementary material for: The Metalloproteinase ADAM28 Promotes Metabolic Dysfunction in Mice
Source: Int J Mol Sci. 2017 Apr 21;18(4):884. doi: 10.3390/ijms18040884 (PMC5412464; doi:10.3390/ijms18040884)
Supplement: Supplementary file 1 [file ijms-18-00884-s001.pdf]

Supplementary Table S1. Calculated T-values.

Figure Number	T-value must be greater than	Calculated T-value	Significance
2	2.064	2.238	Significant
8A	2.776	4.422	Significant
8B	2.776	3.304	Significant
10A	2.447	2.4	Not significant
10E	2.776	3.538	Significant
